# Lecture on Delirium Tremens—Gangrene of the Lungs—Bronchitis—Pulmonary Phthisis

**Published:** 1839-12-14

**Authors:** W. W. Gerhard


### CLINICAL LECTURE.

### PHILADELPHIA HOSPITAL.

*Saturday, December* 7, 1830.

### LECTURE ON DELIRIUM TREMENS—GANGRENE OF THE LUNGS—BRONCHITIS—PULMONARY PHTHISIS.

By W. W. Gerhard, M.D.

*No.* 4—*Winter Course.*

I shall, to-day, continue the subject of the last lecture, viz. phthisis pulmonalis; but, before proceeding to its consideration, I wish to show you a case of *delirium tremens*, or *mania-á-potu.* This is at most times an affection of frequent occurrence; but, for some months, whether from the progressive improvement in the habits of our people, or from other causes, it has become comparatively rare. I submit this patient to your inspection, merely as presenting a good example of the symptoms of *delirium tremens;* reserving the consideration of its pathology and treatment for some future occasion, when I shall detail those changes in the ordinary treatment of the disease which have been made in the hospital within the last few years.

The patient (who has had two attacks of mania-â-potu) was admitted not more than half an hour ago, and has the symptoms of the disease strongly marked. There are tremours over the whole body, and a restless expression of the features. These are the first evident symptoms in nearly every case. But as this case has advanced to the second stage, we have other symptoms, particularly a peculiar disorder of the intellect. The patient offers us an instance of this mental derangement; he is constantly harassed with the fear of being killed by some sudden and violent means, and begs that we may give him time to repent, and not destroy him at once. The hallucination may be of various kinds; but *fear* is one of its most common characteristics. The pupils of both eyes are contracted; the eyes constantly moving in every direction, showing that the muscles of this organ are in the same state of tremulous agitation with those of the rest of the body. The patient is sweating profusely; a circumstance very commonly attending this affection, but not invariably. He has not slept for several nights.

This attack of *mania-â-potu* was the consequence of a fit of drunkenness, which has been almost constant for the last six weeks. Before his admission into the hospital, the treatment consisted in the use of large doses of opium, and emetics. As he seems to be considerably exhausted, I shall order a small quantity of brandy for him, and afterwards put him on the use of Hoffman's anodyne, assafœtida, or some similar antispasmodic. Opium we now very rarely employ; and when we do, we give it in small doses.

I now come to the proper subject of the lecture, and shall present several cases of *phthisis pulmonalis*, and of diseases complicating and simulating it, with a view to the further illustration of its history and diagnosis.

*Case* 1*st.*—You will recollect that at the last lecture I spoke of three modes in which phthisis commences: first, slowly and gradually; secondly, preceded by inflammation; thirdly, commencing with a sudden attack of hæmoptysis. This case is an instance of the third variety of the disease—the *hæmorrhagic.* The patient entered last Saturday; he is a weaver by trade. This employment is a frequent predisposing cause of phthisis, by the circumstances in which the weavers are placed. They work in close rooms, much in the same way as shoemakers and printers, and fatigue excessively the thorax. There are other circumstances which aid in producing this predisposition in weavers; such as long confinement in one position, the dampness of the cellars in which they generally work, and the breathing of an atmosphere loaded with irritating effluvia, which arise from the woollen and cotton goods. The patient had been in good health previously to the present attack; had never had a severe cough for any length of time, and had been temperate in his habits. His parents are still alive and healthy, so that the predisposition is probably not a hereditary one; though instances are occasionally met with in which consumption passes by one generation, and is transmitted from grandfather to grandson, without any indications of its presence at the intermediate point. The account given by the patient of the manner in which he was attacked, is this:—In February last, while engaged at work, he was suddenly seized with spitting of blood; in the course of a fortnight he discharged in this way a very large quantity of blood; he thinks it amounted to six quarts,—but this estimate is, no doubt, much exaggerated. At last he coughed up a large coagulum of blood, by which he was nearly suffocated. From this time the hæmoptysis ceased, but the cough has continued without intermission; the sputa are whitish and muco-purulent. About five months since, he began to be weak and emaciated, and has become more and more so ever since. Chills also commenced with the cough, and have constantly accompanied it up to the present time. This is, therefore, another of those cases of phthisis, which simulate intermittent or remittent fever at their commencement, and of which I have already spoken in the two preceding lectures. The *tubercular fever*, causing this resemblance, occurs in the earlier stages of phthisis, and is truly an irritative disease. Sometimes it assumes the remittent type. I have known two cases of this sort amongst my own acquaintances, which were mistaken for remittent fever, and treated as such; but, after some time, the true nature of the affection was explained, by the clear development of the local signs of phthisis. In the present case there would have been little difficulty in the diagnosis, for the hæmorrhage would have made the character of the fever clear. But, in the majority of cases where hæmoptysis does not precede the tuberculous disease, you will be often aided greatly in your diagnosis by the characters of the fever.

The patient has also suffered from profuse night sweats, and here we have a circumstance which distinguishes this fever from hectic. In the latter, the sweating is not so profuse as in the former; but the chills, on the contrary, are more severe. The patient's appetite has been bad for four or five months; he has had several attacks of diarrhœa, continuing two or three days at a time. Diarrhœa is a common attendant of phthisis pulmonalis, and generally arises from the deposit of tuberculous matter in the small intestines simultaneously with the same process in the lungs; but the frequency and duration of its attacks vary greatly in different cases. You have remarked that the patient was seized with hæmoptysis while at work; it is in this way that it often begins,—suddenly, and while the chest is exposed to some strain.

Hæmorrhage from the lungs, occurring in this sudden manner, is, in most cases, a sure sign of phthisis pulmonalis. It may sometimes arise from other causes, as disease of the heart, congestion of the lungs, or a mechanical cause, such as a strain; but in five cases out of six, in men at least, it is followed by the local signs of tubercular disease. In women, however, it is not so valuable as a diagnostic sign; for in them it may follow suppression of the menses, and may, in fact, become a vicarious discharge. Though hæmoptysis may arise, as I have said, from a sudden muscular effort, when there are no tubercles in the lungs,—yet, in the majority of such instances, they either exist already, or there is a tendency to their formation; for whenever such a tendency exists, the bronchial mucous membrane will bleed from very slight causes. I recollect an instance in which the hæmoptysis was caused by jumping over a wall about five feet in height, and was followed by all the signs of phthisis; and another in which it occurred from lifting a gate which had fallen. The tubercular diathesis, therefore, predisposes to hæmorrhage from the lungs. It is true, that not a few cases of hæmoptysis, abort, as it were, before the tubercles are secreted to any great amount; but when we find that the majority of patients in whom this symptom occurs, are afterwards affected with confirmed phthisis, it is perfectly consistent with the facts to believe that the hæmorrhage coincides with a condition of lungs which favours the tuberculous secretion, and which had in many cases given rise to a limited deposit of tubercle before the discharge of blood took place.

Of the tubercular fever, which is an attendant of the disease in the present case, I have spoken more fully in the preceding lectures. The other general symptoms present nothing peculiar. The emaciation, you perceive, is considerable, and is accompanied by that dirty, earthy hue of the skin, (more especially of the face,) which you have seen in several other patients.

The local signs are—1, cough; 2, expectoration: this at first consisted of mucus, afterwards becoming muco-purulent, with portions of broken down tubercles; 3, dulness on percussion under one of the clavicles, with mucous rhonchi, and a commencing cavernous respiration. These last signs indicate the stage of the disease; they show that there is already some softening of the tubercular matter. This fact is also proved by the character of the sputa.

*Case 2d.*—This patient has been a labourer on the canal. Before the present illness he has never had a cough, or any other sign of pulmonary disease. He has been sick for two years: the attack was gradual, and was produced by taking cold. Since that time there has been constant cough and expectoration; the matter of the sputa is at this time muco-purulent, and somewhat nummular, and begins to be characteristic of the disease. Emaciation has been apparent for eight months: before this attack, the patient was a stout man. The skin is dry, pale, and dusky. There has been one attack of diarrhœa. There has also been fever, with profuse night sweats, but no chills: it is therefore a modified form of hectic. At present there is not much fever—pulse 94. The expectoration consists of round masses floating in a liquid, with small pieces of tubercular matter: at first it was mucous, then muco-purulent. The sputa takes its shape from the cavities in which it is found. We shall presently see that when these cavities are very large, the *nummular* character is no longer present. There has been no hæmoptysis until two nights since, when about a spoonful of blood was discharged. Such small quantities are not important; they may arise from irritation of the bronchial membrane; it is only when hæmoptysis is considerable, that it becomes a sign of importance, and also pathognomonic of phthisis.

*Case 3d.*—This patient was brought before you at the last lecture, as an example of tuberculous disease, coinciding with inflammation of the serous membranes. Since that time, the breathing has continued much oppressed; tongue dry and red; pulse, 95 to 104; bowels constipated. The vesicular murmur has become more distinct on both sides of the chest. There has been an increasing pain in the præcordial regions, with a *bruit de soufflet* of the heart; respiration frequent and high. Yesterday, for the first time; I discovered a grating or creaking sound of the heart, indicative of pericarditis; the *creaking* being produced by lymph into the pericardium. The two surfaces of this membrane rub against each other, chiefly at the beginning of the diastole of the heart, and a grating sound is produced by the spiral movement of the heart on its axis, during the dilatation of the ventricles. The patient, you will recollect, was first attacked by pleurisy; since then, pericarditis has supervened, with more or less endocarditis. This case, is therefore, a good illustration of a circumstance to which I called your attention when I first brought it before you; the connection between phthisis and inflammation of serous membranes. This patient has inflammation of all the serous membranes of the chest, occurring in succession. The pleurisy has declined as the pericarditis has supervened. Besides the signs already mentioned, the existence of pericarditis, attended with effusion, is indicated by feebleness of the impulse of the heart, flatness on percussion, (extending over a larger space than that occupied by the natural dulness of the heart,) and pain on pressure or percussion. The latter symptom, however, is not always present in pericarditis; in any of the serous membranes, in fact, inflammation may occur without any of the ordinary signs. But in this patient the flatness extends to at least double the usual spaces, and there is very decided pain at the region of the heart, which is increased by slight pressure, but is always more or less felt. The dyspnœa in this case is dependant partly on the pleurisy, partly on the pericarditis. The pulse is not invariably altered in inflammation of the heart, or its membranes; it is principally affected in endocarditis, which gives rise to more or less obstruction of the valves. In the present case, there is excitement of the pulse, and slight irregularity.

I shall now show you some cases of gangrene of the lungs, and bronchitis, the symptoms of which more or less resemble those of phthisis, and the diagnosis becomes, therefore, frequently difficult.

Gangrene of the lungs is by no means a frequent disease, it is oftener met with in hospitals than in private practice. It resembles phthisis, inasmuch as it produces softening of the pulmonary tissue, and, consequently the formation of cavities. It differs from it in the fetor of the breath, and expectoration. The local signs, at the commencement of the disease, are imperfect.

The causes of gangrene of the lungs are cold, an epidemic tendency of the atmosphere, intemperance, and depressing circumstances generally. In most cases, it arises from direct exposure, but sometimes it comes on gradually, and appears to be part of a general disease; that is, it depends on a vitiation of the fluids, in the same way with dry gangrene, of which I have shown you an example.

*Case.*—The patient is a boatman forty years of age. He had enjoyed good health till about two months before his entrance into the hospital. At that time, being engaged at his occupation on the Schuylkill, he fell into the river, and was with difficulty saved from drowning. He felt extremely cold, and could not speak for twenty minutes, but no sign of active disease followed for two weeks, other than feebleness and chilliness. Then a cough began, accompanied by pain in the lower part of the right axillary region; the sputa have never contained blood, and have been fetid from the beginning; appetite has been bad throughout; the patient continued to work regularly until November 30th; but since that time, he has been unable to perform any kind of labour. The treatment, previously to his entrance into the hospital, consisted of venesection, and the application of a blister to the right side of the chest.

The patient was admitted December 6th. At that time the symptoms were as follows: slight emaciation; a dusky hue of the skin; slight flushing of the face; dilatation of the nostrils; skin warm; pulse 104, thrilling, moderately resisting; respiration 22, high and laboured; expectoration thick and homogeneous, of a dirty, grayish colour, and very fetid. On the right side, anteriorly, respiration vesicular throughout, with traces of the mucous râle, hurried and harsh at the summit of the lung. On the left side, vesicular, with traces of both mucous and sonorous rhonchi. *Posteriorly*, on the right side, vesicular in upper lobe, hurried, and very feeble; in lower lobe, scarcely any vesicular sound; at the upper part, deep-seated, cavernous respiration, and imperfect pectoriloquy. Percussion gives a flat sound in the lower two-thirds of right side posteriorly; clear anteriorly. The signs, therefore, indicated a cavity in the lower lobe of the right lung, with an engorged condition of the surrounding tissue, accompanied by pleurisy. The treatment has consisted in the use of chloride of soda, given in doses of twenty drops four times a day, with nourishing diet. Quinine, porter, and brandy are often necessary; the indications being to correct the fetor of the breath and expectoration, and support the system, while nature effects the elimination of the gangrenous tissue. A number of palliatives as opiates at night, wilt doubtless occur to you; but you should be sparing of depletory measures; they are rarely necessary, except when there is severe pleuritis near the gangrene; and these should be limited to local bleeding, or still better, to blisters.

Gangrene of the lungs is to be distinguished from phthisis by these circumstances: it usually begins suddenly, and runs its course rapidly; the skin presents a more decided dusky hue in gangrene, than in phthisis; and the breath and expectoration are always fetid from the commencement of gangrene. The prognosis of the two diseases is also very different. In gangrene, it is not necessarily unfavourable; from one-third to one-half of the cases recover; in phthisis, on the contrary, our prognosis is almost always unfavourable after a cavity is formed. When gangrene tends to a favourable termination, recovery generally takes place in a few weeks. Any improvement in the symptoms of phthisis, on the contrary, is very gradually effected.

There are two kinds of expectoration met with in gangrene of the lungs. The most common is blackish, and resembles an inky sediment. The other kind, of which we have an example in the present case, is a grayish, frothy fluid, having some resemblance to yeast, with a fetid odour, which you may perceive is like that of putrid oysters. This, though the least common, is the most favourable variety of sputa. It is generally discharged in very large quantities—amounting, sometimes, to a pint or a quart daily.

I have frequently described, in my lectures, the progress of cure in gangrene. When the sphacelated portion is thrown off, a cavity is formed, lined with the usual pus, secreting false membrane, which gradually assumes the character of a mucous membrane. We shall watch the progress of this case, and keep you informed of the result.

The next case is one of bronchitis. The patient is a labourer, aged 35 years. He entered the hospital on the 2d instant, having been ill for two weeks. He was seized with cough and pain along the sternum; in the course of a week, he began to expectorate a muco-purulent manner, containing no blood; during the most of the time he has been confined to bed. These signs indicate an acute disease, which might be mistaken for the acute form of phthisis. It is distinguished from it, by the absence of the irritable, jerking pulse of phthisis, described in our last lecture, and also, by the absence of the local signs of tubercular deposition. Thus there is no flatness or percussion under the clavicles; and the mucous rhonchus is heard in the sound of respiration throughout the lower lobes of both lungs. But though bronchitis is thus distinguished from phthisis in the commencement, both by the general and local signs, yet it is very apt to terminate in the latter disease, and we ought always to anticipate such a result when it is prolonged, and occurs in young persons.

The next case is a complication of phthisis and bronchitis. The patient is a boatman, 38 years of age, of intemperate habits. He has been sick for three months, and unable to work during the whole of this time; his illness was caused by falling into the canal: the next day he was seized with shivering and cough, unaccompanied by pain: the expectoration consisted of mucus mixed with pus, but no blood. On the 4th instant he entered the hospital, and the symptoms were as follows: There was abundant mucous rhonchus throughout both lungs, passing in certain portions into the sub-crepitant, while at the summit of the left lung, the percussion is dull and the respiration extremely bronchial. There is a quick irritation, some emaciation, and a dry husky skin. The sputa, although not nummular, are more purulent than is usual in cases of bronchitis. The dyspnœa is much greater than in most cases of phthisis or uncomplicated bronchitis.

This case began in the form of bronchitis: phthisis was developed subsequently, and the two diseases are now co-existent. This state of things is of frequent occurrence, particularly at advanced periods of life. At an earlier age, when phthisis is developed in the course of a bronchitis, it is apt to commence more suddenly, and run its course more rapidly than in the present instance. The patient, you perceive, is but slightly emaciated, and will probably get comparatively well: that is, the disease may continue for years, with slight cough, &c., but may not shorten the patient's life; the cavity in the lung remaining, but lined with a healthy membrane. I have known several cases of such comparative recovery, from this form of disease; the chance of long life are not afterwards apparently affected by it.

You will now understand that phthisis pulmonalis may commence in several different forms:

1. It may commence *slowly and gradually.* This is the most common mode of origin, and is generally met with in cases where the tubercular diathesis is hereditary. The first symptoms of the disease are slight cough and expectoration; the local physical signs are not present until a more advanced stage.

2. Phthisis may arise from *inflammation.* This variety is most common in robust persons, and is likewise, in most instances, dependant upon a hereditary predisposition, which imparts to inflammation a tendency to terminate in the formation of tubercles. The most common seat of the inflammation preceding phthisis, is some one or other of the serous membranes; and the tubercles may at first be deposited either in the serous membranes alone, in the lungs, or in both. The mucous membrane of the bronchial tubes may likewise be the seat of the inflammation; but phthisis beginning in the latter way, is more commonly met with in old persons, than that which begins by the serous membranes.

Inflammation performs two distinct parts; in the one it is properly the cause of the tuberculous deposition which may occur some time after the inflammation, or take place during the progress. In the second, the secretion of tubercle is attended with an acute inflammatory action in the organs, but the cause of the tubercles cannot be said to be the inflammation which attends their secretion.

3. The *hæmorrhagic* variety. In this, hæmoptysis, whether preceded by a violent effort or not, constitutes the first symptom.

But these different forms of phthisis, though differing so much in their origin, after a certain period present the same character; they are all attended by emaciation, cough, expectoration consisting of pus and softened tubercular matter, hectic fever, and all the other signs which mark the more advanced stage of the disease. The progress of phthisis is most rapid when produced by inflammation of the serous membranes, especially in young subjects; it is less so when preceded by bronchial inflammation. The hæmorrhagic variety is likewise rapid in its course; the slowest of all is that which is constitutional and hereditary. All of these forms are liable to be confounded with other diseases; thus, the first may be mistaken for simple serous inflammation; the second for bronchitis; the third for hæmorrhage arising from other causes.

We might multiply the varieties of phthisis almost to an indefinite number, but the preceding are the most important, and may be considered as the landmarks in the study of the disease; under one or other of these classes, all other forms may be included. There are likewise other tubercular affections, not commencing in the lungs, and only implicating them secondarily; but phthisis pulmonalis is by far the most frequent form in which the tubercular diathesis develops itself.

I will conclude the lecture by showing you some very interesting pathological specimens, which illustrate this subject. They are the lungs and intestines of a subject who lately died of phthisis in its most aggravated form. I referred to the case at the last lecture; the patient, a young man, being then so feeble as to render it improper to bring him before you. The physical signs, during life, indicated the existence of a large cavity in the left lung: many of you have heard the cavernous, amphoric, and gurgling sounds of respiration which were extremely distinct. In the course of his illness, the patient also had tubercular diarrhœa.

You will at once recognise the existence of a cavity in the upper lobe of the left lung, by the falling in of its parietes as I hold it up. This whole lobe, indeed, is converted into a mere sac, nothing of the normal structure remaining, except the pleura, and a thin layer of the tissue of the lungs on its inner face. The large size of this cavity accounts for the great distinctness of the amphoric respiration in the last stages of the disease. The cavity is lined by a false membrane, and contains a considerable quantity of muco-purulent fluid mixed with particles of tubercular matter. The muco-purulent matter is a secretion from the false membrane; the contents of the cavity differ from the expectoration only, in not containing saliva, which is mixed with them afterwards. The sputa in this case were not of the nummular form of which I showed you a specimen just now, for some days before the death of the patient, because the cavity was too large for their formation. You will notice several bands or bridles passing from one side of the cavity to the other: these consist of blood-vessels, which have resisted the ulceration longer than the surrounding tissue: sometimes, however, they are opened by this process, and hæmorrhage is the result, which is often instantly fatal. The rest of the left lung is infiltrated with grayish tubercular matter to such an extent, that scarcely a trace of the healthy tissue can be found. The tubercles are partly softened, and small cavities are seen here and there: these gave rise to the gurgling sound of respiration.

In the right lung, the lower lobe is in a comparatively healthy condition. The tissue of the upper lobe is engorged with blood: tubercular masses are scattered through every part of it; they are of a yellowish-white colour, and no signs of softening are yet perceptible.

In order that you may see the connection between the lesions and their physical signs, I will read some extracts from the notes of the case. Nov. 4th.—Respiration throughout right side, expansive and full, but a little harsh. Left side, cavernous respiration with distinct pectoriloquy, most evident near the sternum, about the second rib. Nov. 24th.—Anteriorly, very loose gurgling, with cavernous respiration throughout the whole of the left side. Puerile respiration in the right side. Posteriorly, on the left side, very loose mucous rhonchus, with gurgling throughout; but there is a little vesicular murmur near the scapula. Respiration rude in upper third of right lung. 27th.—Left side, anteriorly, respiration amphoric above; loose gurgling in the lower third. Posteriorly (same side,) gurgling and cavernous respiration in the lower half, and at the summit; in the intermediate space, respiration distinctly cavernous, but mixed with a vesicular murmur.

I will now examine the intestinal canal. The mesenteric glands are enlarged, of an irregularly rounded shape, and are entirely converted into tubercular matter. This condition of things, when the tuberculous deposit is confined to the mesenteric glands and adjacent parts, constitutes the disease called *tabes mesenterica.* In most cases of this sort, there are likewise tubercles, either in the peritoneum, or the follicles of the intestine: here they are found in both situations.

*Large intestine.*—In the colon, there are some ulcers in the follicles, with slight inflammation of the mucous coat. Near the rectum are innumerable ulcers of small size, which appear like so many distinct points, because they have commenced in the separate follicles.

*Small intestine.*—Near the ileo-cœcal valve are numerous ulcers, evidently commencing in the glands of Peyer. Some of these glands still remain, but much enlarged, and containing yellowish tubercular matter, which is still of a firm consistence. Here you may distinctly trace the changes which take place in the follicles, from the first separation of the tubercular matter to its complete softening, and final discharge by ulceration. The other viscera were not examined, on account of the short time which remained for us to make the examination previously to the lecture. There is no doubt, however, that tubercles existed in several other organs, particularly the bronchial glands and the spleen, which are amongst the most frequent seats of these deposits.

